# A74 IMPACT OF INDOLE-3-PROPIONIC ACID ON BACTERIAL INVASION POTENTIAL OF *KLEBSIELLA* STRAINS ISOLATED FROM PEDIATRIC ULCERATIVE COLITIS PATIENTS

**DOI:** 10.1093/jcag/gwae059.074

**Published:** 2025-02-10

**Authors:** N Arjomand Fard, H Aujla, L Stuart, C C Cheng, W Elhenawy, T Perry, E Wine

**Affiliations:** University of Alberta, Edmonton, AB, Canada; University of Alberta, Edmonton, AB, Canada; University of Alberta, Edmonton, AB, Canada; University of Alberta, Edmonton, AB, Canada; University of Alberta, Edmonton, AB, Canada; University of Alberta, Edmonton, AB, Canada; University of Alberta, Edmonton, AB, Canada

## Abstract

**Background:**

Indole-3-propionic acid (IPA), a tryptophan metabolite produced by microbes, has demonstrated beneficial effects, including reversing dysbiosis, balancing bacterial populations, and reducing inflammation. However, the mechanisms behind these effects and their impact on pathogenic microbes remain unclear. This study aimed to evaluate the effects of IPA on bacterial invasion *in vitro*, using potential pathobionts (*Klebsiella variicola* and *Klebsiella quasipneumoniae*) isolated from non-inflamed sections of the colon in pediatric ulcerative colitis (UC) patients.

**Aims:**

We hypothesized that IPA could reduce virulence traits, promoting a more homeostatic environment, which could lower inflammation in pediatric UC patients.

**Methods:**

Caco-2 cells (colonic epithelial cell line) were infected with *Klebsiella* strains, with or without 0.5 mM IPA. Bacterial invasion was assessed via gentamicin protection assay, and qPCR measured Interleukin-8, IL-10, and TNF-α expression in infected cells. Transepithelial electrical resistance (TEER) assay evaluated barrier integrity, and qPCR analyzed the expression of bacterial virulence genes, including *entB* (siderophores), *manC* (capsule), and *hcp1* (type VI secretion) in IPA-treated and untreated cultures. Additionally, siderophore production was quantified using a Chrome-Azurol Sulfonate (CAS) assay, and Anthony’s stain visualized the bacterial capsule.

**Results:**

IPA-treated Caco-2 cells showed significantly reduced bacterial invasion, with further reductions when bacteria were also exposed to IPA. qPCR revealed a significant decrease in IL-8 expression in IPA-treated cells during infection. IL-10 levels decreased non-significantly, and TNF-α expression showed variable results. A trend toward improved tight junction integrity was observed in the TEER assay with IPA treatment, although no significant differences were detected. IPA treatment increased the expression of *entB*, *manC*, and *hcp1* genes, except for *K. variicola*, where *manC* expression remained unchanged. IPA significantly reduced the capsule thickness of *K. variicola* and resulted in a significant reduction in *K. quasipneumoniae* siderophore production.

**Conclusions:**

IPA reduces bacterial invasion and modulates immune responses in Caco-2 cells infected with *Klebsiella* pathobiont strains. It decreases the virulence of *K. quasipneumoniae* by targeting siderophore production and diminishes the virulence of *K. variicola* by reducing capsule formation, suggesting its potential as an anti-virulence agent against these pathobionts. These findings highlight IPA’s potential as an immunomodulatory agent for managing IBD. However, further research is needed into other *Klebsiella* virulence pathways and host-microbiota interactions.

**Funding Agencies:**

CIHRWCHRI

